# The Arterial Anatomy of the Cerebellum—A Comprehensive Review

**DOI:** 10.3390/brainsci14080763

**Published:** 2024-07-29

**Authors:** Malwina Błaszczyk, Kajetan Ochwat, Sandra Necka, Maria Kwiecińska, Patryk Ostrowski, Michał Bonczar, Andrzej Żytkowski, Jerzy Walocha, Jerzy Mituś, Mateusz Koziej

**Affiliations:** 1 Department of Anatomy, Jagiellonian University Medical College, Mikołaja Kopernika 12, 33-332 Kraków, Polandsandra.necka@student.uj.edu.pl (S.N.);; 2 Youthoria, Youth Research Organization, 30-363 Kraków, Poland; 3 Department of Anatomy, Faculty of Medicine, University of Social Sciences in Lodz, 90-113 Lodz, Poland

**Keywords:** cerebellum, superior cerebellar artery, anterior inferior cerebellar artery, posterior inferior cerebellar artery, anatomy, aneurysm, stroke, arteries, humans

## Abstract

The cerebellum, a major feature of the hindbrain, lies posterior to the pons and medulla and inferior to the posterior part of the cerebrum. It lies beneath the tentorium cerebelli in the posterior cranial fossa and consists of two lateral hemispheres connected by the vermis. The cerebellum is primarily supplied by three arteries originating from the vertebrobasilar system: the superior cerebellar artery (SCA), the anterior inferior cerebellar artery (AICA), and the posterior inferior cerebellar artery (PICA). However, variations of the cerebellar arteries may occur, such as duplication of the SCA, SCA creating a common trunk with the posterior cerebral artery, triplication of the AICA, and agenesis of PICA, amongst others. Knowledge of the arterial anatomy of the cerebellum is crucial, as inadequate blood supply to this region can result in diminished motor functioning, significantly impacting the quality of life for patients. The present study demonstrated the importance of adequate anatomical knowledge of the arteries supplying the cerebellum. The PubMed and Embase databases were searched to gather articles on the anatomical characteristics and variations of the arterial supply of the cerebellum. It is the most comprehensive and up-to-date review available in the literature. The possible variations of these vessels may be clinically silent or present with clinical symptoms such as neurovascular compression syndromes of the cranial nerves and aneurysms. With a comprehensive understanding of the cerebellar arterial system, physicians can enhance their diagnostic and treatment capabilities, ultimately leading to more effective management of cerebellar vascular-related issues and other neurological deficits.

## 1. Introduction

The cerebellum, a major structure of the hindbrain, lays posterior to the pons and medulla and inferior to the posterior part of the cerebrum. It lies beneath the tentorium cerebelli in the posterior cranial fossa and consists of two lateral hemispheres connected by the vermis [1,2]. The cerebellum is involved in various sensory-motor functions, mainly involving the vestibular and somatosensory systems. Moreover, it may also play a role in some cognitive functions [3]. Knowledge of the arterial anatomy of the cerebellum is crucial, as inadequate blood supply to this region can result in diminished motor functioning, significantly impacting the quality of life for patients [4]. Moreover, the cerebellar arteries have also been related to various neurovascular compression syndromes [5]. Therefore, this comprehensive review aims to provide a better understanding of the general anatomy and variations of the cerebellar arteries.

## 2. Methods

The PubMed and Embase databases were searched to gather articles on the anatomical characteristics and variations of the arterial supply of the cerebellum. The search terms were established from the following phrases and their grammatical variations: ‘cerebellum’; ‘artery’; ‘blood supply’; ‘vascularity’; ‘anatomy’; ‘variation’; ‘morphometry’; ‘type’; ‘superior cerebellar artery’; ‘basilar artery’; ‘pontine artery’; ‘anterior inferior cerebellar artery’; and ‘posterior inferior cerebellar artery’. The terms were individually adapted to each database. Neither date, language, article type, nor text availability conditions were applied. An additional search was conducted through the references of the identified studies at the end of the search stage to ensure the accuracy of the process. The inclusion criteria consisted of original studies with extractable numerical or descriptive data regarding the topic of this study. Articles such as case reports, case series, conference reports, reviews, letters to editors, and studies that provided incomplete or irrelevant data were excluded. Initially, a total of 878 articles were identified. After removing duplicates and excluding irrelevant records, five, seven, and five main studies were selected regarding the superior cerebellar artery (SCA), anterior inferior cerebellar artery (AICA) and posterior inferior cerebellar artery (PICA), respectively. The study collection ended in January 2024.

## 3. Anatomy and Function of the Cerebellum

The cerebellum, meaning “little brain” in Latin, is an important structure in all vertebrates, vital for the coordination of motor movements by integrating sensorimotor information from different areas of the brain and spine [6]. Studies mapping brain connectivity continue to deepen our understanding of the cerebellum’s role in non-motor-related functions such as cognition and emotion [7,8,9,10]. Disruption of the cerebellum’s neural pathways, such as the spinocerebellar tracts (dorsal, ventral, and cuneocerebellar tract), the cerebrocerebellar pathway, and the olivocerebellar tract, may result in a wide range of motor deficits involving eye movement, speech, stance/gait, and limb movements, greatly depreciating the quality of life in affected patients [11].

The cerebellum is situated under the posterior half of the cerebrum and posterior to the brainstem. An extension of the dura mater called the tentorium cerebelli covers the cerebellum, enclosing it within the posterior cranial fossa. It is, more precisely, superior and anterior to the occipital bone, posterior to the brainstem and temporal bones, and inferior to the brain and tentorium. The cerebellum’s cortex is a tightly folded layer of gray matter forming ridges known as folium all over its surface [12]. Beneath the gray matter of the cortex lies the white matter, predominantly composed of myelinated nerve fibers that connect to and from the cortex. Within this white matter, often referred to as the arbor vitae (tree of life) due to its branching, tree-like appearance in cross-section, are four deep cerebellar nuclei consisting of gray matter [13]. From lateral to medial, the deep cerebellar nuclei consist of the dentate, emboliform, globose, and fastigial nuclei. Generally, they receive inhibitory inputs from the Purkinje cells in the cerebellar cortex and excitatory inputs from the climbing fiber and mossy fiber pathways [12].

It is also important to note the presence of the myoclonic triangle, also known as the Triangle of Guillain–Mollaret or the dentato–rubro–olivary pathway, which is a critical feedback circuit involving the brainstem and deep cerebellar nuclei that modulates spinal cord motor activity [14]. It consists of the dentate nucleus, the red nucleus, and the inferior olivary nucleus [15]. Injury to this region can result in several conditions, including hypertrophic olivary degeneration, stroke, brainstem cavernous malformations, palatal tremor, ocular myoclonus, ataxia, nystagmus, and vertigo [15]. Injury to this region can result in several conditions, including hypertrophic olivary degeneration, stroke, brainstem cavernous malformations, palatal tremor, ocular myoclonus, ataxia, nystagmus, and vertigo [16]. 

The shape of the cerebellum reveals roughly three surfaces: the superior or tentorial surface, the petrous surface, and the suboccipital surface, corresponding to the adjoining dura and bones. The cerebellum is divided into the right and left hemispheres, with a midline structure present in the middle known as the vermis. Each cerebellar hemisphere has an anterior, posterior, and flocculonodular lobe separated by fissures [17]. The primary fissure separates the anterior and posterior lobes, the posterolateral fissure separates the flocculonodular and posterior lobes, and the horizontal fissure transects the posterior lobe [2].

Lastly, the cerebellum is intimately related to the brainstem and shares two key structures: the peduncles and the fourth ventricle. The superior, middle, and inferior cerebellar peduncles connect the midbrain, pons, and medulla to the cerebellum, respectively, providing the crucial connection between the cerebellum and the central nervous system for proper neural functioning. The fourth ventricle has a diamond-shaped “floor” on the brainstem’s posterior surface, with the superior and inferior medullary velum as its roof. Therefore, the cerebellar peduncles and the vela are shared by the brainstem and cerebellum [18].

## 4. The Arterial Anatomy of the Cerebellum

**Figure 1:**
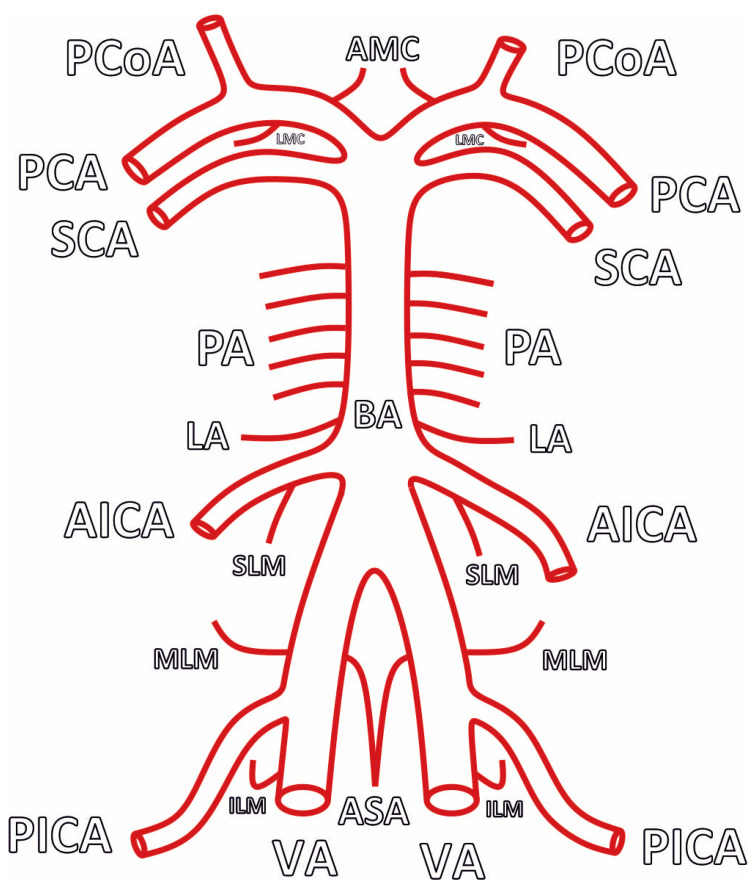
Scheme, illustrating the typical arterial blood supply of the cerebellum. AMC—Anterior Mesencephalic. LMC—Lateral Mesencephalic. PCoA—Posterior Communicating Artery. PCA—Posterior Cerebral Artery. SCA—Superior Cerebellar Artery. PA—Pontine Arteries. BA—Basilar Artery. LA—Labyrinthine Artery. AICA—Anterior Inferior Cerebellar Artery. SLM—Superior Lateral Medullary. MLM—Middle Lateral Medullary. ILM—Inferior Lateral Medullary. ASA—Anterior Spinal Artery. PICA—Posterior Inferior Cerebellar Artery. VA—Vertebral Artery.

**Figure 2:**
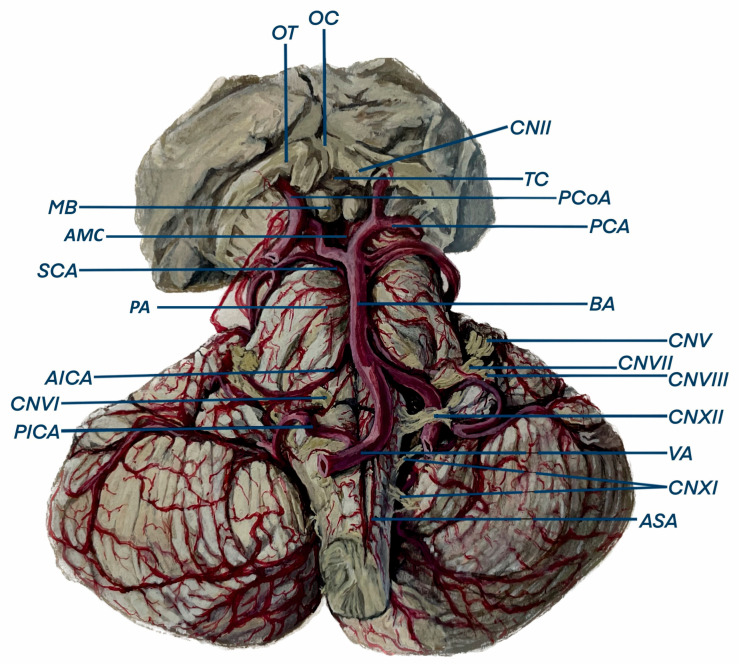
Illustration of the anterior view of the cerebellum in which the duplicated right Superior Cerebellar Artery (SCA) can be observed as well as the right Vertebral Artery (VA) crossing posteriorly the CNXII. MB—Mammillary body. AMC—Anterior Mesencephalic. OT—Optic Tract. OC—Optic Chiasma. CN—Cranial Nerve. TC—Tuber Cinereum. PCoA—Posterior Communicating Artery. PCA—Posterior Cerebral Artery. BA—Basilar Artery. PA—Pontine Arteries. ASA—Anterior Spinal Artery. PICA—Posterior Inferior Cerebellar Artery. AICA—Anterior Inferior Cerebellar Artery.

**Figure 3:**
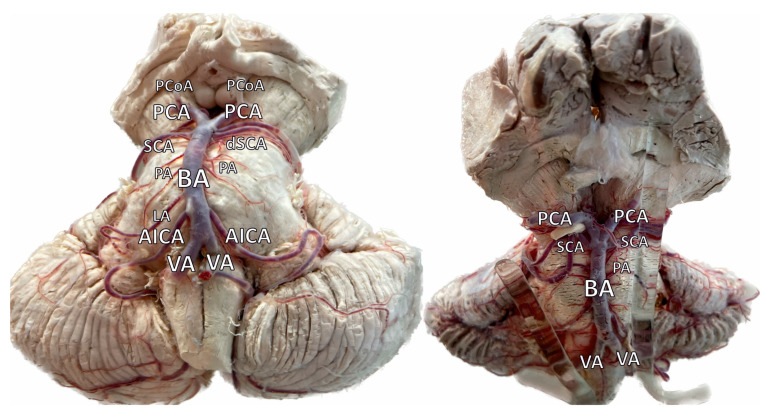
Photos of the cadaveric cerebellum and its arterial supply. PCoA—Posterior Communicating Artery. PCA—Posterior Cerebral Artery. SCA—Superior Cerebellar Artery. dSCA—Duplicated Superior Cerebellar Artery (Left Photo). BA—Basilar Artery. PA—Pontine Arteries. LA—Labyrinthine Artery. AICA—Anterior Inferior Cerebellar Artery. VA—Vertebral Artery. According to the literature, dSCA can be found in 14–35.5% of the general population. Nevertheless, in the presented cadaver, the origin of both dSCA and SCA is typical (from the BA), as it occurs in 90.4–100.00% of the population.

The cerebellum is primarily supplied by three arteries originating from the vertebrobasilar system: the superior cerebellar artery (SCA), the anterior inferior cerebellar artery (AICA), and the posterior inferior cerebellar artery (PICA). They course posteriorly around the brainstem, bifurcating and giving off several branches, and terminate on the posterior-most aspect of the cerebellum. Anastomoses between the cortical segments of the three arteries, consisting of multiple hemispheric branches and some vermian branches, provide an alternative supply and complete the cerebellar arterial supply [19] (Figure 1, Figure 2 and Figure 3). Researchers continue to gradually elucidate the mapping of the cerebellum’s arterial anatomy, with increasing studies being published on the cerebellum and its arterial network [20,21,22].

In “The Cerebellar Arteries” (2000), neurosurgeon Dr. Albert Rhoton Jr. defines three such networks called complexes, which greatly simplify the already complicated anatomy of the cerebellar arteries [19]. The SCA is part of the upper complex. It consists of the mesencephalon, the superior cerebellar peduncle, the cerebellomesencephalic fissure, the oculomotor, trochlear, and trigeminal nerves, and the tentorial surface of the cerebellum. The AICA is part of the middle complex. It consists of the pons, the middle cerebellar peduncle, the cerebellopontine fissure, the abducens, facial and vestibulocochlear nerves, and the petrosal surface of the cerebellum. The PICA is part of the lower complex, which consists of the medulla, the inferior cerebellar peduncle, the cerebellomedullary fissure, the glossopharyngeal, vagal, and accessory nerves, and the suboccipital surface of the cerebellum. The lower complex is often the largest of the three due to the larger blood supply reaching the occipital surface of the cerebellum. However, in some cases, variability in the arterial distribution may render the SCA or AICA as the dominant contributor of blood to the cerebellum.

## 5. The Superior Cerebellar Artery

### 5.1. General Anatomy

The SCA originates from the terminal end of the basilar artery and courses posteriorly around the mesencephalon. It courses past the oculomotor, trochlear, and trigeminal nerves through the cerebellomesencephalic fissure, the superior peduncle, and the tentorial surface of the cerebellum. Moreover, the vessel bifurcates into rostral and caudal trunks, which give off several branches supplying the structures of the upper complex [19]. More specifically, the SCAs primarily supply the upper parts of the vermis, encompassing the central, culmen, declive, and folium lobules. Additionally, they supply the lateral portions of the cerebellar hemispheres, including the anterior, simplex, and superior sections of the semilunar lobules. The SCAs also nourish the cerebellar nuclei—dentate, fastigial, emboliform, and globose—as well as the majority of the cerebellar white matter [23,24]. The SCA is often divided into four segments based on its exact anatomical position: the anterior pontomesencephalic, the lateral pontomesencephalic, the cerebellomesencephalic, and the cortical segments [25]. However, alternative nomenclature related to the ventricular cisterns the artery courses through has been used, namely the ambient and quadrigeminal cisterns referring to the lateral pontomesencephalic and cerebellomesencephalic segments, respectively [26]. Branches of the SCA include the hemispheric, precerebellar, perforating, vermian, and marginal arteries that arise at various locations throughout the course of the artery.

### 5.2. Anterior Pontomesencephalic Segment

The first segment begins with the origin of the SCA from the basilar artery and ends at the anterolateral margin of the pons. The main trunk is easily identified due to its parallel course below the posterior cerebral arteries and the trochlear nerve emerging between them. Its course is caudal and brief, ending at the anterolateral border of the pons near the origin of the trigeminal nerve [27]. Later segments of the SCA course above the trigeminal nerve, and it is no coincidence that this artery is the most common vessel implicated in trigeminal neuralgia due to nerve compression by the vessel [28]. Although approximately one-third of SCAs bifurcate into rostral and caudal trunks in the anterior pontomesencephalic segment, it is not the most common location of bifurcation [29].

### 5.3. Lateral Pontomesencephalic Segment

This segment begins at the anterolateral margin of the pons and ends before it passes into the cerebellomesencephalic fissure. It frequently comes in contact with the trigeminal nerve root as it passes posteriorly. Bifurcation of the SCA most commonly occurs in this segment at approximately 47% to 70% [29]. The trochlear nerve is often in contact with the SCA and has been reported to course most commonly between or inferior to the trunks [29]. This segment gives off a marginal branch to the superior portion of the tentorial surface, making it the first cortical branch of the SCA [19]. The area supplied is inversely related to the area supplied by the AICA, which forms an anastomosis with the SCA at the cortical segment level. The marginal branch also gives perforating branches, which supply the middle cerebellar peduncle and parts of the mesencephalon [19].

### 5.4. Cerebellomesencephalic Segment

This segment begins once inside the fissure and, by extension, the quadrigeminal cistern. It ends upon its exit from the fissure. At this point, the SCA is, in most cases, coursing as the rostral and caudal trunks. The trunks loop sharply within the fissure, giving off precerebellar branches which anchor them to the opposing walls and structures they supply [19]. The trochlear nerve is likewise in contact with the cerebellomesencephalic segment, whose caudal branch intersects the nerve, marking the lateral limit of the quadrigeminal cistern [30].

### 5.5. Cortical Segment

This segment begins once the SCA exits the cerebellomesencephalic fissure and terminates on the superior or tentorial surface of the cerebellum, anastomosing with the cortical segments of the other cerebellar arteries [26]. The cortical segment comprises two groups of branches, the hemispheric and vermian branches, which often consist of several branches themselves. The hemispheric branches arise from the rostral and caudal trunks within the cerebellomesencephalic fissure and supply the superior or tentorial surface of the cerebellum lateral to the vermis. The vermian branches arise from the rostral trunk within the cerebellomesencephalic fissure and supply the superior portion of the vermis. There are commonly two arteries, one bordering the midline strip of the vermis and the other coursing on the paramedian edge bordering the hemisphere [19].

### 5.6. Variations

The SCA’s typical origin, from near the apex of the basilar artery, may be subject to several variations. Due to the proximity of the origin of the posterior cerebral artery (PCA) to the SCA, both may present with a SCA-PCA common trunk variant [31]. The SCA commonly exists as a single vessel present on each side of the cerebellum. However, duplication and triplication of the artery may occur [32]. The SCA is the most consistent of the cerebellar arteries, and its origin remains the main location for variations [33]. The most common variation of the SCA pertaining to its origin is a double origin from the basilar artery, with a prevalence between 14.0% and 35.5%. A duplication variant is similar in that the SCA has two origin sites and continues as separate trunks until they become the rostral and caudal trunks of the normal anatomy. A double origin is different by coalescence into a single trunk [34]. Less common variants include triple origins, total absence of the SCA unilaterally, and even less commonly bilaterally [29] (Table 1).

### 5.7. Clinical Significance

As a major intracranial artery, the SCA may be involved in clinical scenarios such as neurovascular compression (NVC) syndromes and aneurysms [19]. Although normal anatomy may lead to either, anatomical variations have been stipulated to increase the risk of both of the aforementioned pathologies [34]. Surgical procedures of the posterior fossa may also put many vital structures in danger, including the SCA. Therefore, surgeons performing these procedures require comprehensive knowledge and experience to avoid damaging any structures. The SCA’s course passes near the CPA and the nerves of the upper complex, namely the oculomotor, trochlear, and trigeminal nerves. Neural compression syndromes of the oculomotor and trigeminal nerves are well-documented clinical scenarios that may present with symptoms such as oculomotor myotonia or trigeminal neuralgia, respectively [32]. Impingement of the trigeminal nerve by the SCA is the most common cause of trigeminal neuralgia [32]. Variations in the origins of the SCA, such as duplications or increased length and tortuousness, may be involved in NVC syndromes [34]. Asymptomatic cases of impinged nerves by the SCA have also been documented. Avci et al. [35] described the relevance of the SCA caudal and rostral trunks in surgical approaches, namely petrosal, subtemporal, supracerebellar, and infratentorial approaches. However, due to the cranial nerve IV’s proximity and the large number of SCA’s perforating branches, caution and understanding of variations should be exercised when performing such procedures to reduce the risk of possible infarcts or neural damage [29]. With respect to revascularization procedures, the proximal segment of the SCA is an important site for bypasses [26]. Due to the potential occurrence of significant variations of the SCA, physicians performing procedures such as microvascular decompression or aneurysm clipping of the SCA need to perform preoperative imaging to analyze the anatomy of the SCA and look for potential variations in origin or course. Detailed knowledge of SCA variations allows surgeons to plan their approach meticulously, reducing operative time and complications. This can aid in reducing the risk of inadvertent damage to the vessel, subsequently minimizing the risk of postoperative hemorrhage or infarction.

## 6. The Anterior Inferior Cerebellar Artery

### 6.1. General Anatomy

The AICA originates from the inferior half of the basilar artery and courses posteriorly around the pons to supply the cerebellum. The AICA is related to the abducens, facial, and vestibulocochlear nerves, the cerebellopontine fissure, the middle peduncle, and finally, the petrosal surface of the cerebellum together, which constitute the middle complex. It courses over the CPA and commonly bifurcates near the facial-vestibulocochlear nerve complex to form a rostral and caudal trunk. These then give off several branches that supply the structures of the upper complex, including deeper unmentioned neurovascular structures such as deep nuclei and neural tracts [19]. The structures that receive blood from the AICA are the antero-inferior portion of the cerebellum on its petrosal surface, the flocculus, brachium pontis, and the lateral part of the pons [12]. The AICA is commonly divided into four segments: anterior pontine, lateral pontine, flocculonodular, and cortical segments. It also gives off perforating branches, choroidal branches, and unique nerve-related branches, including the labyrinthine, recurrent perforating, and subarcuate branches [19].

### 6.2. Anterior Pontine Segment

The first segment of the AICA begins with the origin, on the basilar artery, and ends at the anterolateral border of the brainstem. It courses caudally as it rounds the pons towards the CPA. It is also usually in contact with the abducens nerve [19].

### 6.3. Lateral Pontine Segment

The lateral segment begins at the anterolateral edge of the pons and terminates at the flocculus. Along the way, it passes the CPA, the facial, and the vestibulocochlear nerves. Most of AICA’s bifurcate into a rostral and caudal trunk before reaching the vestibulocochlear nerve complex [19]. Moreover, the majority of rostral trunks become “nerve-related” and are closely related to the vestibulocochlear nerve complex in proximity and supply. The nerve-related trunk is further divided into premeatal, meatal, and postmeatal parts in relation to the internal auditory meatus [19]. The nerve-related branch of the AICA loops into and out of the auditory meatus, giving rise to the internal auditory arteries, recurrent perforating branches, and subarcuate branches along the way [19]. The former two most commonly arise from the premeatal segment, while the subarcuate branch most commonly arises from the postmeatal segment. The meatal segment forms a convex “medial” loop that, in most cases, is directed towards the auditory canal [19]. A second “subarcuate” loop is sometimes present where the main nerve-related artery forms an “M” shaped loop in the direction of the subarcuate fossa on the temporal bone [19]. The internal auditory arteries, or labyrinthine arteries, enter the auditory canal and reach the inner ear. The recurrent perforating branches course medially to supply the brainstem. The subarcuate branch courses past the subarcuate fossa to reach the subarcuate canal. There are also two cerebellosubarcuate branches, one that courses towards the cerebellum and the other towards the subarcuate canal [36].

### 6.4. Flocculopeduncular Segment

This segment begins just before passing the flocculus and ends just before the SCA appears on the surface of the cerebellum. Most of the AICAs bifurcate into a rostral and caudal trunk, which course above and below the flocculus, respectively. The rostral trunk courses laterally above the flocculus toward the middle cerebellar peduncle, the cerebellopontine fissure, and the adjoining petrosal surface [19]. The caudal trunk courses caudally towards the flocculus and supplies the inferior half of the petrosal surface of the cerebellum and the lateral edges of the fourth ventricle, including the foramen of Luschka and the adjoining choroid plexus [19]. Both enter the cerebellopontine fissure to reach deeper structures, such as the ventricle and the distal parts of the middle peduncle [19].

### 6.5. Cortical Segment

The cortical segment of the AICA begins once the flocculopeduncular segment exits the cerebellopontine fissure, courses onto the surface of the cerebellum, and terminates with distal anastomosis with other cerebellar arteries [19].

### 6.6. Variations

The AICA has several documented cases of variations, most commonly regarding its origin. The normal anatomy of the AICA’s origin includes a single vessel originating bilaterally from the basilar artery and subsequent course towards the cerebellum. Duplication and triplication of this artery have been reported by Martin et al. [36] and Rhoton et al. [19]. Agenesis and hypoplasia of the AICA have also been reported [37]. Approximately 51.3% of AICAs have been documented to originate from the proximal portion of the basilar artery and 48.7% from the middle portion [37]. Interestingly, Kim et al. [38] described a single case, among 48 cadavers studied, of the AICA originating from the vertebral artery. Duplication of AICA was observed in 7.9% of patients in a study of 131 samples conducted by Pekcevik et al. [34], most commonly from the right side. Furthermore, Martin et al. [36] reported duplication of the AICA with a prevalence of 26% in contrast to a single artery with a prevalence of 72% in 50 cerebellopontine angles studied. De Vilalta et al. [39] reported a prevalence of 83.3% single and 16.7% duplication of the AICA. Moreover, Kim et al. [38] reported duplication of the AICA in 4 cases out of 51. Interestingly, Martin et al. [36] and Rhoton et al. [19] both indicate that the AICA rarely presented as a triplicate artery, with a prevalence of only 2%. A prevalent anatomical variation of the AICA is its absence, confirmed by studies performed by Ballesteros et al. [37] and Pekcevik et al. [34], in which agenesis of the AICA, especially on the left side, was noted in 15.2% of 92 cadavers and 36.1% of 131 cadavers respectively (Table 2).

### 6.7. Clinical Significance

The AICA is clinically significant as a major artery of the cerebellum, supplying the pons, middle cerebellar peduncle, and the anterior inferior cerebellum, flocculus, and inner ear [41]. Anatomical variations in the arteries’ course through the CPA and the vestibulocochlear nerve complex may result in NVC syndromes of the nerves of the middle complex. Vascular pathologies such as aneurysms and occlusion may also lead to cerebellar lesions and death, and surgical procedures involving the posterior fossa, CPA, or related structures pose a similar risk if not adequately prepared [32]. Many variations, such as hypoplastic arteries, which may be compensated by the anastomosis of adjacent arteries, are clinically silent [22,42]. Other variations, such as duplications or increased length of the artery, may lead to NVC syndromes, often involving the cranial nerves of the middle complex, namely the abducens, facial, and vestibulocochlear nerves. Compression of the facial-vestibulocochlear nerve complex is most common and may lead to the development of tinnitus, hemiataxia, and hemifacial spasms [36].

Moreover, because the AICA is the main vessel of the CPA, it is at high risk of injury during any surgical procedure in that area, such as excisions of vestibular schwannomas, facial nerve tumors, and arachnoid cysts, amongst others [36,43,44,45]. Knowledge about the anatomical characteristics of the AICA may also be of high significance and bypasses the cerebellar arteries [46]. Due to the clear clinical significance of the AICA, physicians performing procedures associated with this vessel need to be aware of its general anatomical aspects and the numerous variations it may exhibit. Occlusion of the AICA may also lead to lateral pontine syndrome, also known as Marie–Foix syndrome, which is a brainstem stroke syndrome that affects the lateral aspect of the pons [47]. It may affect numerous structures, such as the lateral spinothalamic tract, the facial nucleus, and the principal sensory trigeminal nucleus and tract, amongst others [47].

## 7. The Posterior Inferior Cerebellar Artery

### 7.1. General Anatomy

The PICA is the only cerebellar artery originating from the vertebral artery, not the basilar artery. The artery arises at the level of the medullary olive and passes posteriorly around the medulla through several fissures to terminate on the suboccipital surface of the cerebellum [48]. The PICA is related to the medulla, the glossopharyngeal, vagus, and accessory nerves, the cerebellomedullary fissure, the inferior peduncle, the cerebellar tonsil, the suboccipital hemisphere, and the vermis, all together constituting the lower cerebellar complex. Below the surface, the PICA courses through more than just the cerebellomedullary or tonisllomedullary fissure but also through the telovelotonsillar fissure and the paramedian fissure [48]. The PICA bifurcates like the other cerebellar arteries, except that its trunks are named medial/lateral as opposed to the previous rostral/caudal trunks of the SCA and the AICA. Generally, the medial branch supplies blood to the inferior vermis, including the uvula, nodulus, pyramis, tuber, and in some cases, the declive and the medial portions of the semilunar lobule, gracile lobule, and the tonsil [12,49]. On the other hand, the lateral branch supplies the lower two-thirds of the biventer, the inferior portion of the gracile and semilunar lobules, and the anterolateral portion of the tonsil [12,23]. Lastly, the PICA may also supply the deep cerebellar nuclei, including the fastigial and dentate nuclei [23]. The PICA is commonly divided into the following five segments: the anterior medullary, lateral medullary, tonisllomedullary, telovelotonsillar, and cortical segments [25]. Perforating, choroidal, and several types of hemispheric branches arise from the PICA supplying surrounding structures [19].

### 7.2. Anterior Medullary Segment

The PICA’s first segment originates from the vertebral artery and terminates behind the olive on the border between the anterior and lateral medulla [19]. Since the vertebral artery courses to coalesce in the midline with the contralateral artery to become the basilar artery, the level of origin of the PICA from the vertebral artery determines the presence and length of the anterior medullary segment. An origin point from where the vertebral artery is still on the lateral aspect of the brainstem results in the PICA’s first segment being the lateral medullary segment. The anterior segment gives off perforator branches to the medulla [19].

### 7.3. Lateral Medullary Segment

The lateral segment spans the lateral aspect of the medulla. The rootlets of the facial, vestibulocochlear, and glossopharyngeal nerves emerge here and mark the extent of the lateral aspect of the brainstem and the lateral segment of the PICA. This segment has been reported to the course below, above, and between the rootlets with no prevailing common route [48]. Regarding its common anatomy, the lateral segment is straight or forms a rostral loop and, in a few instances, forms complex loops within the cistern [19]. Furthermore, the lateral segment gives off perforator branches to the medulla.

### 7.4. Tonsillomedullary Segment

The tonsillomedullary segment begins once the PICA is past the rootlets of the glossopharyngeal, vagus, and accessory cranial nerves and courses medially along the posterior aspect of the medulla. The PICA then transitions from the medulla to near the apex of the tonsil, located caudally. The segment forms a caudal loop, called the intratonsillar loop, and ascends the medial aspect of the tonsil until it ends halfway up to become the next segment [19]. By doing so, the segment simultaneously enters the cerebellomedullary fissure. The tonsillomedullary segment gives off choroidal branches supplying the choroid plexus near the fourth ventricle’s midline. In contrast, the choroidal branches of the AICA supply the choroid plexus located laterally, such as near the CPA and the lateral recesses of the fourth ventricle [50]. This segment also gives off perforator branches to the medulla and surrounding structures [19].

### 7.5. Telovelotonsillar Segment

The telovelotonsillar segment begins halfway up the tonsil and continues its course deeper into the cerebellum. The segment exits one fissure by entering another, those being the cerebellomedullary and the telovelotonsillar fissures just beneath the fourth ventricle. The PICA courses to the fissure’s superior extent, where the artery forms a cranial loop and descends to exit the fissures. It gives off branches supplying the choroid plexus like the tonsillomedullary segment, and additionally gives off perforator branches ascending to the dentate nucleus [21]. The segment ends just before its branches turn onto the cortical and vermian surfaces.

### 7.6. Cortical Segments

The cortical segment includes all the trunks and branches of the PICA, which course on the cerebellum’s suboccipital and inferior vermian surface. The cortical branches can be divided into hemispheric, vermian, and tonsillar groups. The medial trunk of the PICA gives off vermian branches, while the lateral trunk gives off hemispheric and tonsillar branches [19]. Usually, one or two vermian branches are coursing in the midline and paramedian fissures. The hemispheric branches course out onto the suboccipital surface of the cerebellum. These branches may range from 0 to 9 with an average of approximately 3, a medial, intermediate, and lateral arm corresponding to the cerebellum’s suboccipital surface [19]. The tonsillar branches supply a portion of the tonsil and, in their absence, are replaced by adjacent hemispheric or vermian branches.

### 7.7. Variations

The PICA exhibits the most tortuous course of the cerebellar arteries and coincidentally contains the most variations [48]. The origin of the PICA has a high prevalence for variations and is the main focus of this subsection. Various positions of the origin of the PICA have been documented, including extradural origins, dual origins, positional variations regarding the vertebral artery, continuation of the vertebral artery itself with ipsilateral hypoplasia, and absence of one altogether [48]. Lister et al. [48], in Microsurgical Anatomy of the Posterior Inferior Cerebellar Artery, documented the position of the origin of 50 samples in regard to eight aspects of the vertebral artery, those being anterior, anteromedial, anterolateral, posterior, posteromedial, posterolateral, and medial and lateral. In order of prevalence, 40% of the PICAs’ origin were located on the posterior aspect of the vertebral artery, 16% on the posterolateral aspect, 16% on the lateral aspect, 6% on the medial, and 2% on the anterior, anteromedial, and anterolateral aspects. None occurred on the posteromedial aspect, and eight PICAs were absent. Furthermore, this study mapped the exact locations of origin in relation to the level of the olive (upper third, middle third, and lower third, and below the lower third of the olive) and in relation to the rostrocaudal line drawn between the medullary pyramids and olive. The results revealed no most common place of origin for the PICA but instead several possible locations, all near the olive. A computed tomography angiography study of 341 patients by Pekcevik et al. [34] revealed approximately 20% of PICAs originating extradurally, 15% originating as continuations of the vertebral artery itself with hypoplasia of the ipsilateral artery, 3% having a double origin, and up to around 40% being nonexistent. The study distinguished the ratios of variants between the right, left, and both arteries. The percentages listed above are the sum of the percentages for each anatomical variant, in this case, pertaining to the origin of the PICA (Table 3).

### 7.8. Clinical Significance

As a major artery of the cerebellum, the PICA is clinically significant in various scenarios. Anatomical variations of the PICA may impinge on the cranial nerves of the lower complex, namely the glossopharyngeal, vagus, and accessory nerves. Surgical approaches to the posterior fossa or other areas near the brainstem and cerebellum need appropriate management to avoid damaging the PICA [46]. Although it is the largest and most variable of the cerebellar arteries, the PICA seldom causes NVC. Instead, it has a higher prevalence for aneurysms due to variations such as extradural and double origins [34], necessitating surgical intervention. PICA aneurysms are most commonly treated surgically or endovascularly [52]. Due to the close proximity of the PICA to the brain stem and the cranial nerves, open surgery may be linked with an increased risk of accidental damage to these structures.

On the other hand, endovascular management of PICA aneurysms has been stated to be a safer treatment option. However, the procedure is highly complicated due to the possible variation in the origin of the artery, as well as because of its tortuous course. Physicians performing this procedure need to have adequate knowledge about the arterial anatomy of the cerebellum. Surgical procedures accessing the posterior fossa require appropriate planning, meaning that the variations of the PICA and other structures should be considered. The PICA is intimately associated with the fourth ventricle and, as such, adds clinical significance due to the latter’s prevalence for other anomalies, such as tumors [53]. The occlusion of PICA may lead to lateral medullary syndrome, also known as Wallenberg’s syndrome [54]. It is characterized by sensory deficits, including contralateral loss of sensation in the trunk and extremities and ipsilateral loss in the face and cranial nerves. Symptoms include pain and temperature sensation loss, ataxia, vertigo with nystagmus, difficulty swallowing, slurred speech, and altered vocal quality. Horner’s syndrome-like symptoms (miosis, anhidrosis, and ptosis) may also occur. Additional signs include palatal myoclonus, hoarseness, nausea, dizziness, bradycardia, and blood pressure changes [5,54].

## 8. Stroke

### 8.1. General Characteristics

Cerebellar stroke accounts for approximately 2% to 3% of all strokes [55]. Due to lower prevalence and nonspecific symptomology, there is a high risk of misdiagnosis and subsequent postinfarction complications. The general symptoms associated with cerebellar stroke include acute headaches, nausea or vomiting associated with vertigo, and ataxia [56,57]. However, the symptoms of acute cerebellar stroke may vary depending on the affected artery.

### 8.2. Superior Cerebellar Artery Stroke

The SCA supplies blood to the superior area of the cerebellar hemisphere, the cerebellar vermis, the superior cerebellar peduncle, the middle cerebellar peduncle, and the interpeduncular region. Moreover, it supplies parts of the midbrain, including the spinothalamic tract, lateral lemniscus, descending sympathetic pathways, and the root of the fourth cranial nerve, amongst others [58,59]. Therefore, occlusion of the SCA may produce various symptoms. The primary cause of SCA occlusion is often attributed to cardioembolisms [60]. In cases of isolated SCA infarctions, the predominant symptoms include headache, gait ataxia, dysarthria, and, less commonly, dizziness and vomiting [60]. Additionally, brainstem findings and hemiparesis have been identified as potential consequences of SCA stroke [60].

### 8.3. Anterior Inferior Cerebellar Artery Stroke

AICA territory infarcts are rare and commonly result in lateral pontine syndrome, or the AICA syndrome [61]. Stroke caused by occlusion of the AICA affects a vast number of nuclei and white matter tracts. These include the facial nucleus, which is specific for AICA infarctions, vestibular nuclei, spinal trigeminal nucleus, spinothalamic tract, sympathetic fibers, middle and inferior cerebellar peduncles, and the labyrinthine artery. Subsequently, the neurological symptoms that may occur due to AICA stroke are facial nucleus effects such as paralysis of the face, decreased lacrimation, taste of the anterior 2/3 of the tongue, salivation, vomiting due to vertigo, nystagmus, contralateral decrease of pain and temperature sensation, ipsilateral Horner syndrome, ipsilateral ataxia and dysmetria, and finally ipsilateral sensorineural deafness [61]. The pathomechanism of AICA stroke has been thought to relate to atherosclerotic occlusion, where atherosclerotic plaques in the basilar artery extend into the origin of the AICA [60].

### 8.4. Posterior Inferior Cerebellar Artery Stroke

PICA stroke is the most prevalent and most studied cerebellar stroke [60,62]. The most typical clinical manifestation of PICA stroke is lateral medullary syndrome, also called Wallenberg syndrome. The dorsal lateral medulla is the most frequently affected ischemic region in PICA stroke, mainly due to the lack of anastomoses between the medullary branches, while the cerebellar and choroidal branches of the PICA have rich anastomoses with the branches of the SCA and the AICA which maintains blood flow to the respective vascular territories [5]. The symptoms specific to PICA infarction occur due to the damage of the nucleus ambiguous (affecting cranial nerves nine, ten, and eleven) and include dysphagia, hoarseness, and decreased gag reflex. Moreover, more symptoms such as vertigo, vomiting, nausea, contralateral loss of pain and temperature sensation, and ipsilateral Horner syndrome may occur due to the damage to the vestibular nuclei, the spinothalamic tract, and the sympathetic fibers, amongst others [60]. The stroke mechanism in cases of PICA occlusion is said to have a cardioembolic or atherothrombotic etiology [60].

### 8.5. Diagnostic Imaging Tools

Magnetic resonance imaging combined with diffusion-weighted imaging is the gold standard for diagnosing cerebellar infarction. This imaging technique enables visualization of impaired blood flow and tissue damage. Furthermore, vascular obstructions may be visualized with magnetic resonance angiography. Unenhanced computed tomography scans are used to assess hemorrhagic lesions and occasionally reveal signs indicative of infarction. However, the effectiveness of computed tomography imaging is hindered by the presence of dense temporal and occipital bones that surround the cerebellum. This reduces its resolution, sensitivity, and specificity compared to MRI, particularly in evaluating cerebellar conditions [63]. On the other hand, when the objective is to analyze the intracranial vascular anatomy, digital subtraction angiography is the gold standard for detailed visualization of the cerebellar arteries. However, the imaging technique is invasive and may cause several complications [64]. Computed tomography angiographies are less invasive and, subsequently, are a safer option [65]. Hence, numerous studies that have analyzed the anatomy of the cerebellar arteries have utilized this imaging modality [34,66,67].

## 9. Conclusions

This study is the most comprehensive and up-to-date review of the cerebellum’s arterial blood supply. Our findings underscore the critical importance of detailed anatomical knowledge of the arteries that supply the cerebellum. Variations in these vessels may be clinically silent or manifest as symptoms like neurovascular compression syndromes of the cranial nerves and aneurysms. We also examined the vascular territories of the SCA, PICA, and AICA, highlighting the potential neurological deficits that can arise from occlusion or damage. A thorough understanding of the cerebellar arterial system can significantly enhance physicians’ diagnostic and treatment capabilities, leading to more effective management of cerebellar vascular-related conditions and other neurological deficits.

## 10. Limitations

The presented study is not without its limitations. The quality and accuracy of a review is directly dependent on the quality of the included studies. Therefore, any limitations resulting from the mentioned articles also apply to the present paper. In this review, relatively few databases were searched to find all articles regarding the studied topic. Furthermore, relatively few studies have investigated and described the anatomical variations of the SCA, PICA, and AICA. This limited literature may cause a gap in the comprehensive understanding of these vascular structures. Further research on the anatomical variations of these structures is needed; however, authors should primarily focus on clinical anatomy, utilizing imaging studies commonly employed in medical practice rather than cadaveric studies. This approach would provide more relevant and practical insights for contemporary clinical applications. Anatomical variations should be furthermore correlated with the clinical condition of the patient.

## Figures and Tables

**Table 1:** Summary of the data from the literature regarding the Superior Cerebellar Artery (SCA). BA—Basilar Artery. PCA—Posterior Cerebral Artery.

SCA Origin	Ballesteros-Acuña 2021 [29]	Banda 2020 [31]	Pekcevik 2013 [34]	Acuña 2001 [35]	Rhoton 2000 [19]
	N = 186	N = 113	N = 341	N = 42	N = 50
Quantity					
Single	74.7%	59.3%	77.5%	67.0%	86.0%
Duplication	22.1%	35.5%	17.6%	26.0%	14.0%
Triplication	0.0%	5.3%	0.0%	7.0%	0.0%
Agenesis	3.2%	0.0%	0.0%	0.0%	0.0%
Origin					
Origin from BA	98.3%	86.8%	90.4%	100.0%	96.0%
Origin from PCA	1.7%	6.2%	4.7%	0.0%	4.0%
SCA-PCA Common trunk	0.0%	7.1%	4.9%	0.0%	0.0%

**Table 2:** Summary of the data from the literature regarding the Anterior Inferior Cerebellar Artery (AICA).

AICA Origin	Ballesteros 2020 [37]	De Vilalta 2019 [39]	Pekcevik 2013 [34]	Rhoton 2000 [19]	Martin 1980 [36]	Nadich [40]
	N = 184	N = 12	N = 341	N = 50	N = 50	-
Quantity						
Single	81.0%	83.3%	56.0%	72.0%	72.0%	60.0%
Duplication	3.8%	16.7%	7.9%	26.0%	26.0%	39.0%
Triplication	0.0%	0.0%	0.0%	2.0%	2.0%	1.0%
Agenesis	15.2%	0.0%	36.1%	0.0%	0.0%	0.0%

**Table 3:** Summary of the data from the literature regarding the Posterior Inferior Cerebellar Artery (PICA).

PICA Origin	Tatit 2022 [21]	Ballesteros 2021 [29]	Pekcevik 2013 [34]	Kawashima 2005 [51]	Rhoton 2000 [19]
	N = 23	N = 186	N = 341	N = 22	N = 50
Single	78.3%	91.4%	61.0%	90.0%	97.6%
Duplication	17.0%	5.7%	0.6%	6.0%	2.38%
Extradural origin	4.3%	N/A	20.8%	N/A	16.7%
Agenesis	-	6.5%	38.4%	4.0%	14.3%
Absent unilaterally	4.3%	N/A	31.7%	N/A	14.3%
Absent bilaterally	8.7%	N/A	6.7%	N/A	0.0%

## Data Availability

The data that support the findings of this study are available from the corresponding author upon reasonable request.
